# Outcomes after Percutaneous Coronary Intervention in Patients with Extremely Calcified Left Main Lesions

**DOI:** 10.3390/medicina59050825

**Published:** 2023-04-23

**Authors:** Silviu Dumitrascu, Daniela Bartos, Claudiu Ungureanu

**Affiliations:** 1 Faculty of Medicine, Carol Davila University of Medicine and Pharmacy, Bvd. Eroii Sanitari 8, 050474 Bucharest, Romania; bartos_daniela@yahoo.co.uk; 2 Cardiovascular Department, Jolimont Hospital, Ferrer St. 159, 7100 La Louviere, Belgium; ungureanu.claudiu@ymail.com

**Keywords:** percutaneous left main revascularization, lesion preparation, calcified lesions, rotational atherectomy, intracoronary lithotripsy

## Abstract

*Background and Objectives:* Available data with regard to the outcomes of patients with severely calcified left main (LM) lesions after revascularization by percutaneous coronary intervention (PCI) when compared to non-calcified LM lesions is unclear. *Materials and Methods:* The present study sought to retrospectively investigate in hospital and 1 year post-intervention outcomes of patients with extremely calcified LM lesions after PCI facilitated by calcium-dedicated devices (CdD). Seventy consecutive patients with LM PCI were included. CdD requirement was based on suboptimal results after balloon angioplasty. *Results:* Twenty-two patients (31.4%) required at least one CdD, while nine patients (12.8%) required at least two. Intravascular lithotripsy and rotational atherectomy were the predominantly used methods(59.1% and 40.9% respectively, for in-group ratios), while ultra-high pressure and scoring balloons contributed the least to lesion preparation (9%). In 20 patients (28.5%), severe or moderate calcifications were angiographically identified, but non-compliant balloon predilation was adequate and CdD were not necessary. Total procedural time was significantly higher in CdD group (*p*-value 0.02). Procedural and clinical success were obtained in 100% of cases. There were no major adverse cardiac and cerebrovascular events (MACCE) recorded during hospitalization. MACCE at 1 year post-procedure were recorded in three patients (4.2% overall). All three events were documented in the control group (6.2%), and no events were recorded in CdD group (*p*-value 0.23). There was one cardiac death at 10 months and two target lesion revascularizations for side-branch restenosis. *Conclusions:* Patients with extremely calcified LM lesions treated by PCI present a favorable prognosis if angioplasty is facilitated by more aggressive lesion debulking using calcium-dedicated devices.

## 1. Introduction

Significant unprotected left main (LM) coronary disease is frequently encountered, with a reported prevalence ranging from 3.6% [1] up to 12% [2] in more comprehensive registries. It represents the subset of coronary lesions with the largest amounts of at-risk myocardium which is concretely manifested as a significant increase in cardiovascular morbidity and mortality when compared to other sites of obstructive coronary artery disease (CAD) [3]. Due to its prognostic implications, revascularization is recommended using a threshold of ≥50% stenosis severity [4,5]. Choosing between PCI or CABG for a particular patient with significant LM CAD is often difficult and heart team interpretation of various clinical and anatomical factors remains of paramount importance.

Coronary artery calcification (CAC) represents a frequent finding in patients with CAD [6]. Even with notable advancements in the interventional field, patients with moderate-to-severe CAC are still considered to have a worse prognosis after PCI when compared to patients without calcifications [6,7,8]. Those with severe calcifications of LM lesions are often excluded or underrepresented in studies, making it difficult to extrapolate currently known outcomes for this subpopulation of patients. Up to now, the available data are not entirely clear concerning the outcomes of patients with severely calcified LM lesions after revascularization by PCI when compared to non-calcified LM lesions. As previously mentioned, LM PCI creates an inherently worse prognosis in the medium to long-term when compared to non-LM PCI [3]. Similarly, the presence of CAC at any site may lead to unsatisfactory procedural results, thereby creating a worse prognosis [6,8]. Accordingly, in patients with LM CAD, the presence of significant calcifications at the target lesion can often lead to technical difficulties and a negative impact on prognosis; conceivably, these calcifications could represent a critical factor for a revascularization strategy that favors CABG over PCI.

Similar to severe CAC at any location, LM lesion preparation using calcium-dedicated devices (CdD) is known to be both feasible and technically effective. Nonetheless, in real-world practice, a notable level of reluctancy might be observed with regard to CdD employment in LM located lesions. This can be explained by several factors, including the higher-risk location, increased complexity due to the presence of a critical bifurcation, insufficient operator expertise, higher costs, and lack of dedicated studies to validate the (long-term) risk-benefit ratio.

Thus, the present study sought to retrospectively investigate the in hospital and 1 year post-procedure clinical outcomes of patients with extremely calcified LM lesions after PCI facilitated by CdD.

## 2. Methods

### 2.1. Design and Patients

We retrospectively included all consecutive patients who underwent LM PCI at a single center in Belgium during a 12 month period (January 2021 to December 2021). Outcomes were evaluated during index hospitalization and at 12 months post-procedure using data from in hospital visits, telephone interviews (if clinical follow-up was not performed), or national data registries for death occurrence, if necessary, in particular cases. The study included a total of 70 patients. All the LM procedures were performed by a single experienced operator. There were no exclusion criteria.

Significant LM CAD was defined as ≥50% diameter stenosis in any segment of the LM. Other vessel significant CAD was defined as stenosis of >70% or 40–70% with invasive or non-invasive proof of ischemia. Protected LM was defined when at least one patent graft to the left coronary system was present. Ad-hoc procedures were performed in patients with acute coronary syndrome and ongoing ischemia. Patients with stable clinical status and high anatomical complexity were discussed in the local heart team. The decision to perform either PCI or CABG was based on anatomic complexity, surgical risk, comorbidities, the possibility of maintaining dual-antiplatelet therapy for at least 6 months, and patients’ informed preference.

The diagnostic and PCI procedures were performed as per conventional and local practices. During the PCI procedure, unfractionated heparin was administered to obtain an activated clotting time of 250–300 s. The antiplatelet regimen included administering aspirin with a thienopyridine at the operator’s discretion (75 mg of clopidogrel, 180 mg of ticagrelor, or 10 mg of prasugrel) for a period of at least 12 months. The type of stent, intravascular imaging, circulatory mechanical support, and the intraprocedural usage of GP IIb/IIIa antagonists were all left at the discretion of the operator.

As per local protocols, all LM lesions were predilated before stent implantation using non-compliant (NC) balloons (with or without semi-compliant (SC) balloon dilatation as a first step). Insufficient plaque preparation was considered, by visual inspection, as >20% under-expansion of a 1:1 sized NC balloon (angiographically and by stent enhancement if available). In the scenario of insufficient plaque preparation, additional plaque modification maneuvers were used according to operator judgement: scoring balloon, cutting balloon, ultra-high-pressure balloon (OPN^®^, SIS Medical AG, Frauenfeld, Switzerland), intravascular lithotripsy (IVL), or rotational atherectomy (RA). RA was also utilized as a primary strategy in cases where the operator’s judgement deemed initial balloon dilatation as futile. NC balloon post-dilatation was used at the discretion of the operator. Procedural details of the CdD techniques are described in the Supplementary Data.

Given the current limitations of establishing calcification severity by angiographic means [9], it was determined that assigning the interest group by angiographic calcification severity was inadequate for two reasons: (1) the existence of moderately calcified lesions that could require a CdD; (2) the existence of severely calcified lesions in which NC balloon dilation leads to an adequate lesion preparation, with no CdD necessary. Hence, the interest group in the present study (i.e., extremely calcified—CdD Group) was defined by the employment of CdD as per the operator’s judgment. The authors acknowledge the possible inclination to consider this a rather biased approach in group assignment; however, this was considered to be a strategy that is more closely related to real-world clinical scenarios.

### 2.2. Data Collection

Patients were stratified according to the necessity to perform additional plaque modification maneuvers using CdD for the LM lesions. The the study population was divided into a group of extremely calcified LM lesions (CdD Group) and a second group used as control (regular LM PCI—rLM Group). It should be noted that the dividing factor—necessity of CdD—was independent of the degree of calcification severity. as assessed angiographically.

### 2.3. Definitions

Procedural success was defined as final residual diameter stenosis <20% with TIMI 3 flow. Clinical success was defined as procedural success and hospital discharge without death, stroke, or urgent repeat revascularization. Death was classified as either cardiac (myocardial infarction, heart failure, arrythmia) or non-cardiac (confirmation of any other cause). For myocardial infarction (MI), the fourth universal definition was used [10]. Peri-procedural MI was defined as highly-sensitive troponin elevation >3× the baseline regardless of electrocardiogram or echography ischemic changes. Target lesion revascularization (TLR) was defined as revascularization by either PCI or CABG, performed for the treatment of in-stent restenosis (ISR) or stent thrombosis (ST) within 5 mm of the stent, involving either the main branch or the side branch. ST was defined according to Academic Research Consortium [11]. Major adverse cardiac and cerebrovascular events (MACCE) were defined as the presence of at least one of the following: cardiac death, non-procedural myocardial infarction, TLR, or stroke. Bleeding events were defined according to current recommendations [12]. In-hospital morbidity was defined as access site-related bleeding, non-access site related bleeding, or acute kidney injury occurring before discharge. MACCE was used as primary endpoint, while its components, procedural, and clinical success were all used as secondary endpoints.

The SYNTAX Score was used to quantify the anatomical severity of the lesions [13]. Disease distribution in bifurcation lesions was described using Medina classification [14]. Coronary lesion calcification as per angiography were defined as moderate (radiopaque densities only during the cardiac cycle and involving only one side of the vascular wall) or severe (radiopaque densities seen before contrast injection independently of the cardiac motion).

### 2.4. Statistical Analysis

Categorical data are shown as absolute and relative frequencies. Continuous variables are shown as mean ± standard deviation if normally distributed, or as median and interquartile range (IQR) if non-normally distributed. Normality tests were performed using histograms and the Shapiro–Wilk test. Inter-group comparison for categorical variables was performed using chi-square test (or Fisher’s exact test, as appropriate). For continuous data, Student’s *t* or Mann–Whitney–Wilcoxon U test were used. All the reported *p*-values are two-sided, and values of <0.05 were considered significant. Data analysis was performed using IBM SPSS Statistics for Windows, version 20 (IBM Corp., Armonk, NY, USA).

## 3. Results

### 3.1. Baseline Population Characteristics

During the analyzed period, a total of 870 PCI procedures were performed at our center, of which 70 involved LM angioplasty and were included in the present analysis. The baseline characteristics of the study population are presented in Table 1. Twenty-two patients (31.4%) required CdD and were included in the CdD Group (72.7% males, 68.64 ± 7.63 years of age); the remaining 48 patients were included in the rLM Group (70.8% males, 65.58 ± 11.29 years of age). There were no significant differences between groups with regard to demographic data, the presence of cardiovascular risk factors, history of coronary revascularization, or chronic kidney disease. Stable angina represented the primary clinical presentation in both groups (68.2% CdD vs. 54.2% rLM, *p* = 0.49). Multivessel coronary disease was a frequent finding in both groups, with three-vessel disease observed in 14 (63.6%) in the CdD Group and 26 (54.1%) in the rLM Group (*p* = 0.20). The mean SYNTAX score for the entire population was 28.45 ± 8.89, with no significant difference between the two groups (29.18 ± 9.92 CdD vs. 28.11 ± 8.47 rLM, *p* = 0.84). The total number of patients with SYNTAX score >32 was 22 (31.4%), with numerically more in the CdD group (36.3%) when compared to the rLM group (29.1%; *p* = 0.17).

### 3.2. Procedural Details

Procedural details and angiographic characteristics with inter-group comparisons are detailed in Table 2. A minority of the procedures were performed ad-hoc, with significantly less in the CdD Group (13.6% vs. 33.4%, *p*-value = 0.03). Overall, the preferred access site was radial in 61 patients (87.1%) from the total population, with no significant differences between groups. Distal radial access was performed in 45 (64.2%) of the total cases, and the usage of CdD created no significant difference with regard to the distal approach. A sheathless vascular system was used to facilitate transradial access in 47 cases (67.1%), of which 36 cases required a 7F Sheathless system. Distal radial access was performed exclusively using the RailTracking technique, as was previously described [15]. Pharmacological support before procedure was required in two cases (2.9%), and one patient required mechanical circulatory support (MCS) before the procedure; all three were part of the rLM group.

LM stenosis ≥ 70% was observed in 36 patients (51.1%) of the total population, numerically more in the CdD Group (59.1% vs. 47.9%, *p* = 0.38). The ostium of the LM was found to be involved in two cases (9.1%) in the CdD Group, and four cases (8.3%) in the rLM Group (*p* = 0.91). There were no calcified lesions observed angiographically in two patients from the CdD Group, while severe and moderate calcifications were identified in four (8.3%), and sixteen (33.3%), respectively, in the rLM Group.

Overall, new-generation drug eluting stents were implanted in 69 patients (98.6%), while a drug-eluting balloon was used in one case (1.4%) as treatment for LM in-stent restenosis. LM non-bifurcation stenting was performed in one case (1.4%). The up-front two-stents technique was used 17 cases (24.3%) from the total (intergroup comparisons and detailed techniques used are shown in Table 3).

There were no significant differences between the two groups with regard to the LM stent diameter (3.55 mm ± 0.21 CdD vs. 3.59 mm ± 0.26 rLM, *p* = 0.40), nor for the LM stent length (37.27 mm ± 17.54 CdD vs. 36.11 ± 15.79 rLM, *p* = 0.86). However, there was a significant tendency for increased LM final stent diameter through proximal optimization technique (POT) balloon diameter in the CdD Group (5.41 ± 0.36 CdD vs. 5.18 ± 0.44 rLM, *p* = 0.03). Overall, final kissing balloon optimization was performed in 67 cases (95.7%). Only one case with provisional strategy required side-branch (SB) bail-out stenting.

**Figure 1:**
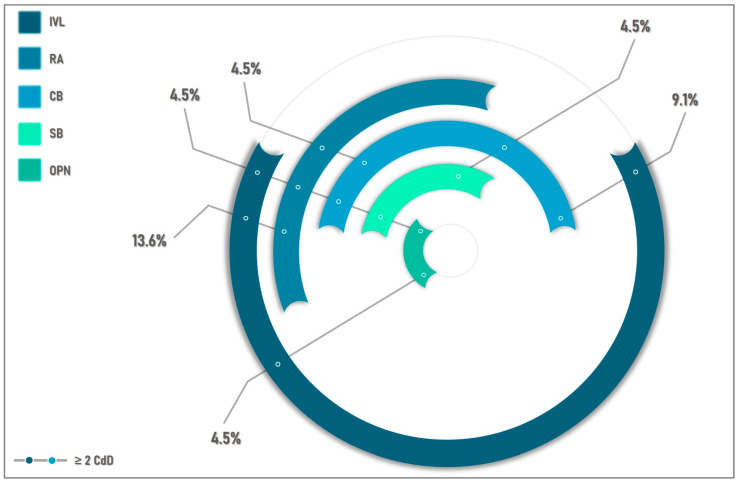
**CdD Group—Visual depiction of the usage of dedicated devices and their synergistic combination.** IVL was the most used technique (63.6% in total; 27.2% of cases as the single CdD), followed by RA (40.9% in total; 18.1% of cases as the single CdD), and cutting balloon (31.8% in total; 13.6% of cases as the single CdD). Scoring and OPN balloons were each used in 2.8% of cases, only in association with other CdD. In 40.9% of cases, a synergistic combination of at least two CdD was necessary; the greatest utility was observed with RA followed by IVL (13.6%). CB—cutting balloon; CdD—Calcium-dedicated device; IVL—intravascular lithotripsy; RA—rotational atherectomy; SB—scoring balloon; OPN—Super high-pressure balloon.

For the CdD Group, calcium-modifying techniques are shown in Figure 1 and technical details are displayed in Table 4. RA was used in nine cases (12.9% from total population), among which five were found to have insufficient lesion preparation after RA, and further lesion preparation was employed. IVL was used in three cases for additional lesion preparation after RA, and in another three cases for additional preparation after either cutting balloon or OPN. In six cases (8.5% from total population), IVL was used as a single CdD and proved to be sufficient for lesion preparation before stent implantation. One patient required RA followed by scoring and cutting balloons, IVL, and OPN in order to obtain a satisfactory lesion preparation. A cutting balloon was used in three cases (4.2%) and prepared the lesion appropriately unaided by other CdD (Figure 1).

Complete revascularization during index procedure was achieved in 53 patients (75.7%), with no significant differences between groups (77.3% CdD vs. 75% rLM, *p* = 0.83). Multi-vessel PCI was performed during index procedure for 41 patients (58.6%), with at least one other complex lesion (B2/C by ACC/AHA classification) treated in 23 patients (32.8%), with no significant differences between groups (Supplementary Table S1). Chronic total occlusion (CTO) PCI was performed during index procedure in four cases (5.7% from the total population). The total non-LM stent length during index procedure was, on average, 44.56 mm ± 21.9 in the CdD Group, and 51.10 ± 33.79 in the rLM Group (*p* = 0.85).

Intravascular imaging was used in five cases from the CdD group (22.7%) and nine cases (18.8%) in the rLM group (*p* = 0.69). Details with regard to the procedural phase in which intravascular imaging was used were not recorded, nor were the morphological lesion details. No additional steps were required for stent optimization following intravascular imaging.

During index procedure, a total of two complications occurred, both in the rLM Group. Longitudinal compression of the LM stent occurred during LM PCI in one patient (treated with repeated POT), and coronary balloon perforation during non-LM vessel PCI in one patient (treated successfully with covered stent). Procedural success was obtained in all patients.

### 3.3. In-Hospital Events

Clinical success was obtained in all 70 cases (100%). During index hospitalization, the incidence of in-hospital morbidity was 2.9% (two cases): one case of minor gastro-intestinal bleeding in the CdD Group, and one case of contrast-induced nephropathy in the rLM Group. Both cases were managed with a conservative approach. In-hospital outcomes are shown in Table 5.

### 3.4. Follow-Up Clinical Events

Clinical data and patient status at 12 months were available for 68 patients (97.1%). Survival status at 12 months was available for 70 patients (100%). Clinical events at follow-up are described in Table 6. Primary endpoint of MACCE occurred in 4.2% (three patients) from the total, with all the events occurring in the rLM group (6.2%) (Supplementary Table S2). Survival-free rate at 1 year was 98.5% for the entire study population, with one death occurring at 10 months in a patient from the rLM group. The patient died shortly after being admitted to the hospital for heart failure presented as cardiogenic shock.

Elective coronary angiography was performed if required by symptoms, proof of myocardial ischemia at non-invasive testing, or based solely on physician’s judgment as related to index procedure established risk (in conformity with the local protocols for high-risk procedures). Accordingly, repeat coronarography was available 43 patients from the entire study population (61.4%), with 15 patients from the CdD group (68.1%) and 28 patients from the rLM group (58.3%; *p* = 0.18). Median time to repeat angiography was 44.4 weeks with no significant differences between groups. Relevant findings at repeat angiography are shown in Table 6. SB restenosis was found in two patients from the rLM Group (4.1%). De-novo significant lesions unrelated to index procedure were found in two patients (one from each group; 4.5% and 2%, respectively, *p* = 0.87). There were no statistically significant differences between groups with regard to the outcomes (Table 6). Kaplan–Meier curves and log-rank tests for comparison were ultimately considered ineffectual due to the unexpected low rate of events at follow-up.

## 4. Discussion

Several key points are to be taken from the present analysis: (i) patients with extremely calcified LM lesions appeared to have a good prognosis after PCI facilitated by CdD; (ii) aggressive lesion preparation strategies appear safe, as they added no notable immediate complications when compared to standard lesion preparation; (iii) the overall incidence of adverse clinical outcomes in the total LM population was unexpectedly low at 1 year post-procedure when compared to available data in the literature.

The existence of calcifications in LM location represents a strong predictor of mortality [16]. Additionally, when PCI is also performed in this subset of patients, the acute mechanical result has a significant influence on distant-term outcomes [7,17], suggesting the importance of plaque preparation.

Available data for proper interpretation of our results is scarce and consists of mostly retrospective studies or post-hoc analyses from several registries. Regarding RA performed for severely calcified LM lesions, a post-hoc examination of the ROTATE registry revealed an in-hospital MACE rate of 5.8%, and 26.4% rate at 12-months follow-up, driven mostly by TVR (20.3%) [18]. These results are in line with several observational studies that focused strictly on the outcomes of patients with LM disease in which RA was used for debulking; these studies revealed in-hospital MACCE rates between 5.8% and 13.4%, with a 12 month rate of MACE or TLR ranging between 13.3 and 26.4% [19,20,21,22,23,24,25]. A recent post-hoc analysis from the EXCEL trial evaluated the clinical outcomes of the included patients according to lesion preparation strategies [26]. Herein, RA and cutting/scoring balloons (CSB) were used in 6% and 9.5% of cases, respectively, and they were compared with the groups in which either an NC balloon was used for lesion preparation or direct-stenting was performed. The RA group presented the highest rate of procedural complications (16.2%), although not statistically significant by comparison. The 3 year rate of MACE showed no difference between groups (RA 17.9%, 20.2% CSB, 14.5% NC, 14.7% direct stenting, *p* = 0.50); however, a higher numerical tendency of stent thrombosis was noted in the RA and CSB groups [26].

The available data are even less robust when it comes to other CdD used in LM disease. Lesion preparation with scoring or cutting balloons in LM calcified lesions is proved to be feasible, but available data on clinical impact are limited [27,28]. Concerning shockwave IVL for LM lesion preparation, some case reports are available [29,30], in addition to some small retrospective studies in which the follow-up period was short, revealing a rate of MACE events of 12.5% and 3.2% at 30 days follow-up [31,32]. Recently, a direct comparison between RA and IVL in LM lesions was published [33]. There were no statistically significant differences between the two groups with regard to outcomes, and it revealed a MACE rate in accordance to previously mentioned studies (in hospital 10.3% for RA, 6.7% for IVL; at 6 months 17.2% for RA, 13.3% for IVL) [33].

The present analysis might be regarded as revealing a more favorable clinical outcome of this high-risk patient population with extremely calcified LM lesions in which at least one dedicated device was required. There were no events recorded during hospitalization or at 1 year follow-up in the CdD Group. In comparison to what is currently described, the results become additionally relevant and they can be explained by several factors. First, in our study there was possibly a lower threshold for the use of CdD. This would result in a better plaque preparation if compared to exclusively NC balloon preparation. These additional procedural steps might have determined a better stent apposition and higher final stent areas. Additionally, 40% of the patients in this group had plaque preparation with more than one dedicated device. The algorithm used in the selection of the CdD was based on operator preference, anatomical features, plaque morphology, and anticipated deliverability. It is noticeable that RA and, in particular, Shockwave IVL represented the main pillars in the CdD group (either individually or in combination), with only a small gap outside the RA-IVL scope (Figure 1). The high utility of RA and IVL can be explained by their individual effectiveness, their combined efficacies [34], and by the LM plaque characteristics which morphologically could require more aggressive preparation when compared to other coronary locations [35].

Furthermore, in the present analysis there were no significant device-related complications that otherwise might have offset the outcomes. One other notable aspect was the significant tendency towards higher POT balloon diameter in the CdD group. A final stent area obtained in these patients could partly explain the better outcomes.

The overall MACCE event rate for the entire LM population in our study was 4.2% at 12 months post-procedure. This is substantially less that what was anticipated using available data from LM PCI RCTs [36,37,38,39]. This should be interpreted in the context of a small number of patients included in our analysis. Nevertheless, the low rate of events in the present study can be related to several factors: (i) the type of stents used (exclusively new-generation DES with high radial force, particularly in the CdD group); (ii) the systematic approach to pre-dilation and post-dilation regardless of the angiographic calcification; (iii) repeat final POT following each additional maneuver capable of stent deformation; (iv) side-branch optimization and final kissing balloon dilation; and (v) low residual SYNTAX scores. In addition, given that all the procedures were performed by a single operator, the present analysis might have naturally benefited from a more uniform and consistent approach as opposed to previously published multi-center, multi-operator trials.

Several studies demonstrated the positive clinical impact that IVUS has in LM PCI [40,41,42,43]; consequently, an indication of class IIA currently exists for IVUS in this context [4,5]. In IVUS-guided LM PCI, better long-term outcomes have been consistently ascribed to a greater final stent area [40,41,43]. Aside from this factor, other technical features correlated, to some degree, with better outcomes derived from IVUS: the stent post-dilation in itself, higher post-dilation balloon diameter, higher post-dilation pressure, longer stents, and more complete revascularization [41,43]. Thus, according to existing data, we should consider a greater final stent diameter as a marker of a more successful LM PCI procedure, as indicated by the IVUS-dictated propensity to increase post-dilation balloon size in order to correct angiographically-guided balloon selections.

In the present analysis, the usage of intravascular imaging was relatively low (20% of cases); as such. the final clinical outcomes cannot be significantly attributed to imaging. Nevertheless, it becomes noticeable that our technical approach bears resemblance to IVUS-related approaches in which high(er) balloon sizes were used for post-dilation [40,41,43]. From our data (Table 3—data available for 95.7% of the patients), if we use the MB and SB balloons diameters as references, the anticipated POT balloon diameter for LM—as per the established laws of fractal geometry [44]—should have been at a mean average of 4.1 mm. However, the final POT balloon diameter in our data stood at a mean average of 5.1 mm, with a mean average of differences equal to 0.81 mm between what it could have been considered proper angiographically and what was actually performed. Thus, there exists the probability of having obtained a greater final stent area, and it may seem reasonable to assume that an additional benefit related to IVUS would not have been as large as it was in other studies with regard to stent optimization [40,41,43].

One other important feature of our study is related to the high use of transradial approach, and particularly distal radial approach (in 64.2% from the total procedures) using the RailTracking technique [15]. The rate of distal radial approach was numerically higher in the CdD Group, showing the lack of significant impediments in using this approach even when aggressive debulking is required. Thus, a high rate of radial approach allowed for properly-sized systems associated with a possible decrease in the incidence of access-site related complications.

Completeness of revascularization is a known prognostic factor after PCI, particularly in the case of three-vessel or LM CAD [45,46]. In the studied population, a residual SYNTAX score of 0 was obtained in 75.7% of cases. During the index procedure, non-LM PCI contributed to the completeness of revascularization in nearly 60% of cases, among which almost a third were complex lesions, indicating the feasibility of performing additional angioplasty during index procedure in favor of the objective to obtain a null or minimal residual SYNTAX score.

Limitations of this study are first related to the small sample size. In addition, the absence of a methodical pre-specified plan for the employment of a particular CdD (or their association) could be considered as another limitation. The 1 year outcomes data period might be regarded as insufficient, and a threshold of 3 or 5 years could be more reliable.

## 5. Conclusions

Patients with extremely calcified LM lesions treated by PCI present a favorable prognosis if angioplasty is facilitated by more aggressive lesion debulking using CdD. A lower threshold for device employment and their association did not represent a trigger for significant peri-procedural adverse events when compared to conventional PCI. Clinical outcomes at 1 year post-procedure were similar between groups and presented with a low incidence of adverse events overall. The results might contribute to the difficult decision-making process regarding the risks and benefits of using debulking tools for optimal plaque preparation in LM PCI.

## Figures and Tables

**Table 1:** General characteristics of the study population. Values are presented as *n* (%), mean ± SD, median (IQR).

	Total *n* = 70	CdD *n* = 22	rLM *n* = 48	*p*-Value
Age	66.54 ± 10.32	68.64 ± 7.63	65.58 ± 11.29	0.28
Male sex	50 (71.4%)	16 (72.7%)	34 (70.8%)	0.87
Diabetes	26 (37.1%)	8 (36.4%)	18 (37.5%)	0.92
Insulin	8 (11.4%)	2 (9.1%)	6 (12.5%)	0.67
BMI	29.27 ± 5.22	30.68 ± 5.14	28.63 ± 5.19	0.13
Hypercholesterolemia	69 (98.6)	21 (95.5%)	48 (100%)	0.13
Arterial hypertension	63 (90%)	20 (90.9%)	43 (89.6%)	0.86
Peripheral Arterial Disease	9 (12.9%)	4 (18.2%)	5 (10.4%)	0.36
Malignancy	8 (11.4%)	3 (13.6%)	5 (10.4%)	0.69
Active smoker	16 (22.9%)	3 (13.6%)	13 (27.1%)	0.21
CrCl < 60 mL/min/1.73 m^2^	6 (8.6%)	2 (9.1%)	4 (8.3%)	0.91
Dialysis	1 (1.4%)	0	1 (2.1%)	0.49
Previous PCI	22 (31.4%)	10 (45.4%)	12 (25%)	0.30
Previous CABG	0	0	0	
LVEF (%)	49 ±7.1	48.41 ± 6.43	49.38 ± 7.55	0.34
Clinical Presentation				0.49
Stable angina	41 (58.6%)	15 (68.2%)	26 (54.2%)	
Non-STE ACS	17 (24.3%)	4 (18.2%)	13 (27.1%)	
STEMI	3 (4.3%)	0	3 (6.3%)	
Silent ischemia	9 (12.9%)	3 (13.6%)	6 (12.5%)	
PCI in previous 30 days	7 (10%)	3 (13.6%)	4 (8.3%)	0.98

ACS—acute coronary syndrome; BMI—body mass index; CABG—coronary artery bypass graft; CrCl—creatinine clearance; LVEF—left ventricular ejection fraction; PCI—percutaneous coronary intervention; STEMI—ST segment elevation myocardial infarction.

**Table 2:** General procedural and angiographic details. Values are presented as *n* (%), mean ± SD, median (IQR).

	Total *n* = 70	CdD *n* = 22	rLM *n* = 48	*p*-Value
Timing				0.03
Ad-hoc	19 (27.1%)	3 (13.6%)	16 (33.3%)	
Elective	51 (72.9%)	19 (86.4%)	32 (66.7%)	
SYNTAX Score	28.45 ± 8.89	29.18 ± 9.92	28.11 ± 8.47	0.84
SYNTAX Score >32	22 (31.4%)	8 (36.3%)	14 (29.1%)	0.17
Pharmacological support before procedure	2 (2.9%)	0	2 (4.2%)	0.49
MCS before procedure	1 (1.4%)	0	1 (2.1%)	0.49
Type of MCS	ECMO	n/a	ECMO	
Transradial	61 (87.1%)	19 (86.3%)	42 (87.5%)	0.52
Distal radial	45 (64.2%)	16 (72.7%)	29 (60.4%)	0.42
Sheathless system				0.34
Total	47 (67.1%)	16 (72.7%)	31 (64.5%)	
7F	36 (51.4%)	14 (63.6%)	22 (45.8%)	
6F	11 (15.7%)	2 (9.1%)	9 (18.8%)	
Catheter size				0.19
7F	59 (84.3%)	19 (86.3%)	40 (83.3%)	
8F	1 (1.4%)	1 (4.5%)	0	
Branching				0.87
Bifurcation	67 (95.7%)	22 (100%)	45 (93.8%)	
Trifurcation	3 (4.3%)	0	3 (6.3%)	
LM stenosis ≥70%	36 (51.4%)	13 (59.1%)	23 (47.9%)	0.38
Three-vessel disease	25 (35.7%)	8 (36.3%)	17 (35.4%)	0.94
Distal bifurcation angle				0.48
<45°	3 (4.3%)	0	3 (6.3%)	
45–70°	27 (38.6%)	9 (40.9%)	18 (37.5%)	
>70°	40 (57.1%)	13 (59.1%)	27 (56.3%)	
LM ostial involved	6 (8.6%)	2 (9.1%)	4 (8.3%)	0.91
LM body shaft only	5 (7.1%)	1 (4.5%)	4 (8.3%)	0.56
Medina Classification				0.61
1,1,0	39 (55.7%)	10 (45.5%)	29 (60.4%)	
1,1,1	17 (24.3%)	6 (27.3%)	11 (22.9%)	
1,0,1	6 (8.6%)	3 (13.6%)	3 (6.3%)	
1,0,0	6 (8.6%)	3 (13.6%)	3 (6.3%)	
0,0,1	1 (1.4%)	0	1 (2.1%)	
Angiographic LM calcification				0.02
No/mild	30 (42.8%)	2 (9.1%)	28 (58.3%)	
Moderate	23 (32.9%)	7 (31.8%)	16 (33.3%)	
Severe	17 (24.2%)	13 (59.1%)	4 (8.3%)	
Eccentric calcification	19 (27.1%)	7 (31.8%)	12 (25%)	0.55
Lesion thrombus	3 (4.3%)	0	3 (6.3%)	0.23
LM in-stent restenosis	1 (4.3%)	0	1 (2.1%)	0.49

LM—left main; MCS—mechanical circulatory support.

**Table 3:** Left main angioplasty—procedural details. Values are presented as *n* (%), mean ± SD, median (IQR).

	Total *n* = 70	CdD *n* = 22	rLM *n* = 48	*p*-Value
LM only PCI	29 (41.4%)	9 (40.9%)	20 (41.7%)	0.95
Stent LM towards				0.14
CX	6 (8.6%)	3 (13.6%)	3 (6.3%)	
LAD	64 (91.4%)	19 (86.4%)	45 (93.8%)	
LM pharmacological balloon	1 (1.4%)	0	1 (2.1%)	
LM non-bifurcation stenting	1 (1.4%)	0	1 (2.1%)	0.49
Bifurcation 1-stent	51 (72.9%)	16 (72.7%)	35 (72.9%)	0.98
Bifurcation 2-stents	17 (24.3%)	6 (27.3%)	11 (22.9%)	0.69
2-stent technique				0.54
DK-Crush	11 (15.7%)	4 (18.2%)	7 (14.6%)	
TAP	3 (4.3%)	1 (4.5%)	2 (4.2%)	
Culotte	1 (1.4%)	0	1 (2.1%)	
Nano-Crush	2 (2.9%)	1 (4.5%)	1 (2.1%)	
LM pre-dilation	70 (100%)	22 (100%)	48 (100%)	
Pre-dilation balloon size	3.19 ± 0.35	3.09 ± 0.33	3.24 ± 0.35	0.13
Pre-dilation pressure	20.1 ± 2.2	21.2 ± 2.5	18.8 ± 1.9	0.08
Pre-dilation SB	17 (24.3%)	6 (27.3%)	11 (22.9%)	0.74
LM stent diameter	3.58 ± 0.24	3.55 ± 0.21	3.59 ± 0.26	0.4
SB stent diameter	3.01 ± 0.32	2.96 ± 0.33	3.05 ± 0.33	0.66
LM stent length	36.48 ± 16.25	37.27 ± 17.54	36.11 ± 15.79	0.86
POT balloon diameter	5.25 ± 0.43	5.41 ± 0.36	5.18 ± 0.44	0.03
Tri-kissing	2 (2.9%)	0	2 (4.2%)	0.28
SB stenting required	1 (1.4%)	0	1 (2.1%)	0.56
SB bailout stenting technique	T-stenting	n/a	T-stenting	
Covered LM ostium	67 (95.7%)	21 (95.5%)	46 (95.8%)	0.94
Final KBD	67 (95.7%)	22 (100%)	45 (93.8%)	0.23
MB KBD diameter	3.70 ± 0.24	3.7 ± 0.27	3.70 ± 0.23	0.93
SB KBD diameter	3.02 ± 0.36	2.99 ± 0.43	3.04 ± 0.32	0.35
Re-POT	17 (24.3%)	6 (27.3%)	11 (22.9%)	0.69
Stent Name				0.02
Synergy Megatron^TM^ (Boston Sci.)	39 (55.7%)	18 (81.8%)	21 (43.8%)	
Xience^TM^ (Abbott)	29 (41.4%)	4 (18.2%)	25 (52.1%)	
Promus Elite^TM^ (Boston Sci.)	1 (1.4%)	0	1 (2.1%)	
Angiographic success	70 (100%)	22 (100%)	48 (100%)	
Complications during LM PCI	1 (1.4%)	0	1 (2.1%)	0.56
Type of complication	Stent longitudinal compression—1 (1.4%)	n/a	Stent longitudinal compression—1 (2.1%)	
Treatment of complication	Re-POT—1 (1.4%)	n/a	Re-POT—1 (2.1%)	
Pharm. support initiated during procedure	0	0	0	
MCS initiated during procedure	0	0	0	
Procedure time (s)	76.54 ± 36.5	93.18 ± 42.29	69.92 ± 31.15	0.02
Fluoroscopy time (s)	1579 ± 686.9	1865.18 ± 699.8	1448.19 ± 646.65	0.16
Total dose area product (mGy.m^2^)	8.73 ± 6.32	8.83 ± 5.8	8.69 ± 6.24	0.5
Contrast amount (mL)	168.57 ± 60.56	170.14 ± 69.88	167.85 ± 56.57	0.89
Guide extension	7 (10%)	6 (27.3%)	1 (2.1%)	<0.001
Microcatheter	5 (7.1%)	3 (13.6%)	2 (4.2%)	0.31
Intravascular imaging				
Total	14 (20%)	5 (22.7%)	9 (18.8%)	0.69
IVUS	10 (14.3%)	4 (18.2%)	6 (12.5%)	
OCT	4 (5.7%)	1 (4.5%)	3 (6.3%)	
LM MSA (mm^2^)	12.4 ± 2.1	12.8 ± 1.7	11.5 ± 2.8	0.24
Complete revascularization	53 (75.7%)	17 (77.3%)	36 (75%)	0.83
No. of other significant lesions	2 (1–2.25)	2 (1–2)	2 (1–3)	0.4
Any CTO	9 (12.9%)	4 (18.2%)	5 (10.4%)	0.36
Planned PCI for residual lesions	14 (20%)	6 (27.2%)	8 (16.6%)	0.21
Residual SYNTAX Score ≥8	6 (8.5%)	1 (4.5%)	5 (10.4%)	0.15

CTO—chronic total occlusion; CX—circumflex artery; DK—double kissing; IVUS—intravascular ultrasound; KBD—kissing balloons dilation; LAD—left anterior descending artery; LM—left main; MB—main branch; MCS—Mechanical circulatory support; MSA—minimal stent area; OCT—optical coherence tomography; PCI—percutaneous coronary intervention; POT—proximal optimization technique; SB—side branch; TAP—T-and-protrusion.

**Table 4:** Calcium-dedicated devices usage. Values are presented as *n* (%), mean ± SD, median (IQR).

	Total *n* = 70	CdD *n* = 22	rLM *n* = 48
Ca-dedicated devices	22 (31.4%)	22 (100%)	n/a
Rotational atherectomy	9 (12.9%)	9 (40.9%)	n/a
Bailout RA	5 (7.1%)	5 (22.7%)	n/a
Max. burr size	1.75 (1.5–2)	1.75 (1.5–2)	n/a
No. of burrs			
1 burr	8 (11.4%)	8 (36.3%)	n/a
2 burrs	1 (1.4%)	1 (4.5%)	n/a
Burring LM towards			
LAD	8 (11.4%)	8 (11.4%)	n/a
CX	1 (1.4%)	1 (1.4%)	n/a
IVL	13 (18.5%)	13 (59.1%)	n/a
IVL balloon size	3.41 ± 0.49	3.41 ± 0.49	n/a
No. of pulses on LM	56.92 (30–80)	56.92 (30–80)	n/a
IVL device crossing success	n/a	100%	n/a
IVL used in >1 segment	10 (14.2%)	10 (45.4%)	n/a
Cutting Balloons	7 (10%)	7 (31.8%)	n/a
Scoring Balloon	2 (2.8%)	2 (9%)	n/a
OPN Balloon	2 (2.8%)	2 (9%)	n/a
OPN/IVL for stent under-expansion	0	0	n/a

Ca—calcium; CX—circumflex artery; IVL—intravascular lithotripsy; LAD—left anterior descending artery; LM—left main; RA—rotational atherectomy.

**Table 5:** In-hospital events and discharge treatment. Values are presented as *n* (%), mean ± SD, median (IQR).

	Total *n* = 70	CdD *n* = 22	rLM *n* = 48	*p*-Value
**In-hospital**				
Procedural success	70 (100%)	22 (100%)	48 (100%)	n/a
Clinical success	70 (100%)	22 (100%)	48 (100%)	n/a
Death before discharge	0	0	0	n/a
Peri-procedural MI	0	0	0	n/a
In-hospital morbidity	2 (2.8%)	1 (4.5%)	1 (2.1%)	0.87
Bleeding	1 (1.4%)	1 (4.5%)	0	
Access site related complication	0	0	0	
Contrast induced-nephropathy	1 (1.4%)	0	1 (2.1%)	0.566
Complication treatment	Conservative	Conservative	Conservative	
MACCE	0	0	0	n/a
**At-discharge**				
Aspirin	70 (100%)	22 (100%)	48 (100%)	n/a
Thienopyridine	70 (100%)	22 (100%)	48 (100%)	n/a
Clopidogrel	42 (60%)	14 (63.6%)	28 (58.3%)	
Ticagrelor	27 (38.6%)	8 (36.4%)	19 (39.6%)	
Prasugrel	1 (1.4%)	0	1 (2.1%)	
Beta-blocker	45 (64.3%)	10 (45.5%)	35 (72.9%)	0.02
ACEi	58 (82.9%)	17 (77.3%)	41 (85.4%)	0.40
Statins	67 (95.7%)	20 (90.9%)	47 (97.9%)	0.17

ACEi—angiotensin-converting enzyme inhibitors; MACCE—major adverse cardiovascular and cerebral events; MI—myocardial infarction.

**Table 6:** Events at 12-months follow-up. Values are presented as *n* (%), mean ± SD, median (IQR).

	Total *n* = 70	CdD *n* = 22	rLM *n* = 48	*p*-Value
MACCE	3 (4.2%)	0	3 (6.2%)	0.23
Cardiac death	1 (1.4%)	0	1 (2.1%)	0.49
Non-procedural MI	0	0	0	n/a
TLR	2 (2.8%)	0	2 (4.1%)	0.33
Stroke	0	0	0	n/a
All-cause death	1 (1.4%)	0	1 (2.1%)	0.49
Survival at 1-year	69 (98.5%)	22 (100%)	47 (97.9%)	0.49
MB in-stent restenosis	0	0	0	n/a
SB restenosis	2 (2.8%)	0	2 (4.1%)	0.33
Possible ST	1 (1.4%)	0	1 (2.1%)	0.49
Hospitalization for HF	1 (1.4%)	0	1 (1.4%)	0.49
Bleeding	4 (5.7%)	2 (9%)	2 (4.1%)	0.41

HF—heart failure; MACCE—major adverse cardiovascular and cerebral events; MB—main branch; MI—myocardial infarction; SB—side branch; ST—stent thrombosis; TLR—target lesion revascularization.

## Data Availability

The datasets analyzed during the current study are available from the corresponding author on reasonable request.

## Supplementary Materials

The following supporting information can be downloaded at: https://www.mdpi.com/article/10.3390/medicina59050825/s1. Table S1. Non-LM PCI performed during index procedure.; Table S2. Individual MACCE description.
