# Supplementary material for: Outcomes after Percutaneous Coronary Intervention in Patients with Extremely Calcified Left Main Lesions
Source: Medicina (Kaunas). 2023 Apr 23;59(5):825. doi: 10.3390/medicina59050825 (PMC10222037; doi:10.3390/medicina59050825)
Supplement: Supplementary file 1 [file medicina-59-00825-s001.zip › medicina-2336021-supplementary.pdf]

Supplementary Data

Rotational atherectomy procedure (ROTAPRO™, Boston Scientific) was performed using a burr-to-artery ratio of ≤0.5. The rotational speed was used inside the range of 160,000 and 180,000 rotations per minute, and speed decrements of more than 5000 rpm were to be avoided. The burr was positioned immediately proximal to the lesion to avoid injury to the healthy vessel segment. Heparin, verapamil, and nitroglycerin was administered during RA as an intracoronary infusion, and a temporary pacemaker wire was to be inserted during RA of the right coronary artery and the left circumflex artery in patients with a dominant left system *only* in the case of clinically significant and sustained bradycardia.

Intravascular lithotripsy with Shockwave™ Catheter (Shockwave Medical Inc.) – a target balloon-to-artery ratio of 1:1 was used. The balloon was positioned at the lesion level and pulses were emitted with the balloon inflated at 4 atm, followed by transient inflation to nominal pressure of 6 atm for the assessment of lesion preparation. If the lesion was uncrossable to the IVL catheter, adjunctive methods were allowed at the operator's discretion (buddy wire technique, pre-dilatation with a smaller diameter NC or compliant balloon, guide catheter extension).

Table S1. Non-LM PCI performed during index procedure. Values are presented as n (%), mean ± SD, median [IQR]

Table 1	Total n=70	CdD n=22	rLM n=48	p-value
Non-LM PCI performed	41 (58.6%)	13 (59.1%)	28 (58.3%)	0.95
No. of non-LM lesions treated	2 [1-2]	2 [1-2]	2 [1-2]	0.82
No. of non-LM vessels treated	1 [1-2]	1 [1-1.5]	1 [1-2]	0.69
No. of non-LM stents	1 [1-2]	1 [1-2]	2 [1-2]	0.75
Involved main territories				0.12
Left	35 (50%)	13 (59%)	22 (45.8%)	
Both	4 (5.7%)	0	4 (8.3%)	
Right	2 (2.9%)	0	2 (4.2%)	
≥1 B2/C Lesions*	23 (32.8%)	9 (40.9%)	14 (29.2%)	0.07
≥1 bifurcation PCI	16 (22.9%)	6 (27.3%)	10 (20.8%)	0.55
CTO PCI	4 (5.7%)	1 (4.5%)	3 (6.3%)	0.77
Conventional PCI	31 (44.3%)	9 (40.9%)	22 (45.8%)	0.7
Maximum Stent diameter	3.27 (0.52)	3.39 (0.35)	3.21 (0.58)	0.44
Total non-LM Stent Length	49.13 ± 30.48	44.56 ± 21.90	51.10 ± 33.79	0.85
Complications	1 (1.4%)	0	1 (2.1%)	0.49
Type of complication	Balloon perforation - 1 (1.4%)	n/a	Balloon perforation - 1 (2.1%)	

**Interventional treatment of complication**	Covered Stent - 1 (1.4%)	n/a	Covered stent - 1 (2.1%)
**Angiographic success**	100%	100%	100%

CTO – chronic total occlusion; LM -left main; PCI – percutaneous coronary intervention.
*ACC/AHA classification.

Table S2. Individual MACCE description.

	MACCE	Time to MACCE	Age	SS	rSS	CdD	LVEF	Initial presentation	Procedure	Non-LM PCI
**Patient 1**	Cardiac death	10	74	33	0	No	30%	Silent ischemia	Provisional stenting	Conventional
**Patient 2**	SB restenosis	12	55	29	0	No	55%	STEMI	2-stent strategy (TAP)	Conventional
**Patient 3**	SB restenosis	12	58	18	0	No	55%	Non-STE ACS	Provisional stenting	Provisional

CdD – calcium-dedicated devices; LVEF – left ventricular ejection fraction; LM – left main; MACCE - major adverse cardiovascular and cerebral events; PCI – percutaneous coronary intervention; rSS – residual SYNTAX Score; SB – side branch; STEMI – ST elevation myocardial infarction; SS – SYNTAX Score; TAP – T-and-protrusion
